# An orthorhombic polymorph of mulinic acid

**DOI:** 10.1107/S1600536810000528

**Published:** 2010-01-09

**Authors:** Iván Brito, Jorge Bórquez, Luis Alberto Loyola, Matías López-Rodríguez, Alejandro Cárdenas

**Affiliations:** a Departamento de Química, Facultad de Ciencias Básicas, Universidad de Antofagasta, Casilla 170, Antofagasta, Chile; b Instituto de Bio-Orgánica ’Antonio González’, Universidad de La Laguna, Astrofísico Francisco Sánchez N°2, La Laguna, Tenerife, Spain; c Departamento de Física, Facultad de Ciencias Básicas, Universidad de Antofagasta, Casilla 170, Antofagasta, Chile

## Abstract

The title compound [systematic name: (3*S*,3a*S*,10b*R*)-3-isopropyl-5a,8-dimethyl-2,3,4,5,5a,6,7,10,10a,10b-deca­hydro-*endo*-epidioxy­cyclo­hepta­[*e*]indene-3a(1*H*)-carboxylic acid], C_20_H_30_O_4_, is a polymorphic form of a previously reported structure [Loyola *et al.* (1990 ▶). *Tetra­hedron*, **46**, 5413–5420]. The newly found ortho­rhom­bic polymorph crystallizes in *P*2_1_2_1_2_1_ with two mol­ecules in the asymmetric unit. The mol­ecules are linked into discrete *D*(2) chains by simple O—H⋯O inter­actions. There are only slight variations in the mol­ecular geometry and supra­molecular organization in the crystal structures of the two polymorphs. The densities are 1.145 (monoclinic, *P*2_1_) and 1.155 Mg m^−3^ (ortho­rhom­bic, *P*2_1_2_1_2_1_).

## Related literature

For background to the structures of mulinic acid, see: Loyola *et al.* (1990 ▶, 2004 ▶). For their biological activity, see: Munizaga & Gunkel (1958 ▶); Araya *et al.* (2003 ▶). For related structures, see: Brito *et al.* (2008*a* ▶,*b* ▶). For puckering parameters, see: Cremer & Pople (1975 ▶). For hydrogen-bond motifs, see: Bernstein *et al.* (1995 ▶).
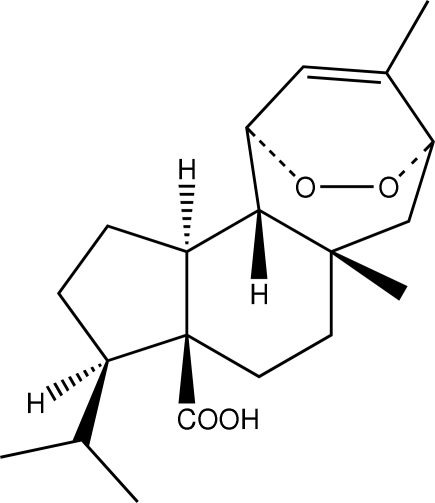


## Experimental

### 

#### Crystal data

C_20_H_30_O_4_
*M* *_r_* = 334.44
Orthorhombic, 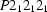

*a* = 7.4160 (15) Å
*b* = 19.374 (4) Å
*c* = 26.767 (5) Å
*V* = 3845.8 (13) Å^3^
*Z* = 8
Mo *K*α radiation
μ = 0.08 mm^−1^
*T* = 293 K
0.18 × 0.15 × 0.08 mm

#### Data collection

Nonius KappaCCD area-detector diffractometer
43215 measured reflections
5387 independent reflections
4452 reflections with *I* > 2σ(*I*)
*R*_int_ = 0.08

#### Refinement

*R*[*F* ^2^ > 2σ(*F* ^2^)] = 0.091
*wR*(*F* ^2^) = 0.224
*S* = 1.25
5387 reflections
444 parameters
H-atom parameters constrained
Δρ_max_ = 0.45 e Å^−3^
Δρ_min_ = −0.48 e Å^−3^

### 

Data collection: *COLLECT* (Nonius, 2000 ▶); cell refinement: *DENZO-SMN* (Otwinowski & Minor, 1997 ▶); data reduction: *DENZO-SMN*; program(s) used to solve structure: *SIR97* (Altomare *et al.*, 1999 ▶); program(s) used to refine structure: *SHELXL97* (Sheldrick, 2008 ▶); molecular graphics: *ORTEP-3 for Windows* (Farrugia, 1997 ▶) and *PLATON* (Spek, 2009 ▶); software used to prepare material for publication: *WinGX* (Farrugia, 1999 ▶).

## Supplementary Material

Crystal structure: contains datablocks global, I. DOI: 10.1107/S1600536810000528/om2310sup1.cif

Structure factors: contains datablocks I. DOI: 10.1107/S1600536810000528/om2310Isup2.hkl

Additional supplementary materials:  crystallographic information; 3D view; checkCIF report

## Figures and Tables

**Table 1:** Hydrogen-bond geometry (Å, °)

*D*—H⋯*A*	*D*—H	H⋯*A*	*D*⋯*A*	*D*—H⋯*A*
O2*A*—H2*AA*⋯O1^i^	0.82	1.86	2.681 (4)	177
O2—H2⋯O1*A*^ii^	0.82	1.89	2.702 (4)	175

Symmetry codes: (i) 
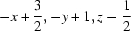
; (ii) 
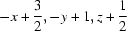
.

**Fig. 1.**
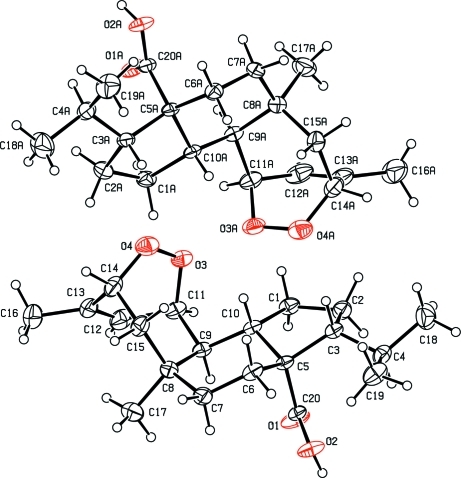
A view of the molecular structure of the title compound, with the atom numbering scheme. Displacemenent ellipsoids are drawn at the 30% probability level and H atoms are shown as small spheres of arbitrary radii.

**Fig. 2.**
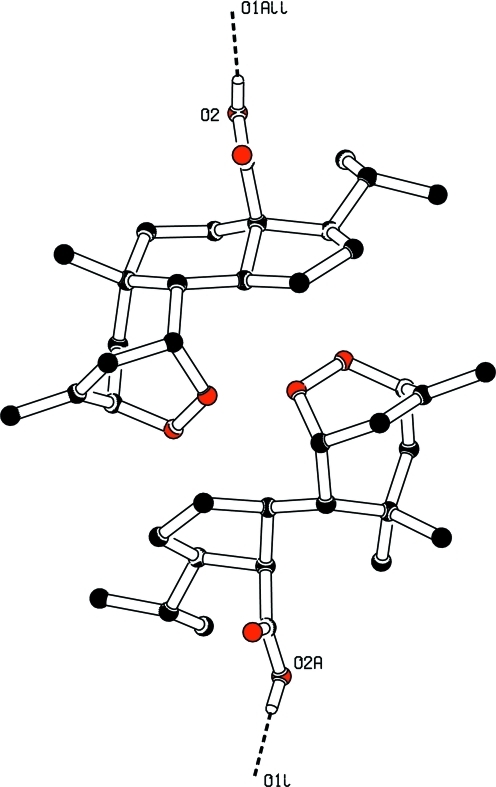
Part of the crystal structure, showing the discrete chain D(2) motif. For clarity only the H atoms involved in the hydrogen bonds (dashed lines) are shown.

## supplementary crystallographic information

### Comment

Polymorphism in crystalline material may occurs as a result of crystallization from different solvents, crystallizations in the presence of small-molecules or macromolecular additives, or phase transitions *etc* (Bernstein *et al.*, 1995). We have obtained an orthorhombic modification of (I) by crystallization from a chloroform-acetonitrile (1:1) whereas the monoclinic phase was obtained from ethylacetate-n-hexane solution of (I) (1:1). The title compound was obtained from dried and finely powdered aerial parts of Mulinun crassifolium Phil. (Loyola *et al.*, 1990).This plant, commonly know as chuquican, susurco or espinilla, is used in folk medicine, principally against diabetes, and bronchial (cough) and intestinal disorders (Munizaga & Gunkel, 1958). Mulinane diterpenes exhibit antiplasmodial and anti-tripanosomacruzi (Araya *et al.*, 2003) activity. We report here the crystal structure of a new polymorphic form of a previously reported structure, characterized by different crystal packing according to our X-ray investigation and a lower melting point than the reported for the known monoclinic phase (132–139° *vs* 185–187°, respectively). We were not able to determine the absolute stereochemistry by X-ray methods, and the configuration shown here was chosen to be in accord with the reported in the previous X-ray diffraction studies (Loyola *et al.* 2004). The molecule of the title compound is built up from three fused carbocycles, one five-menbered, one six-membered and one seven-membered ring. The five- membered ring has an envelope conformation (ring A) whereas the six-membered ring has a perfect chair conformation (ring B) and the seven-menbered ring has a boat conformation (ring C) respectively. [Q_2_=0.442 (4) Å, φ =104.8 (6)°; Q_T_=0.556 (4) Å, θ=163.6 (4)°, φ =180.4 (2)°; Q_T_=1.153 (4) Å, φ_2_ =179.5 (2)°] (Cremer & Pople, 1975). The isopropyl, methyl groups and the carboxylic acid at C3, C8 and C5a are β-oriented respectively, whereas the *endo*-peroxide group is α-oriented. The molecules are linked into *R*^2^_2_ (8) dimers by simple O–H···O interactions (Table 1 and Fig. 2). There are only slight variations in the molecular geometry and supramolecular organization in the crystal structures of the two polymorphs. The densities are 1.145 Mg m^-3^ (monoclinic, P2_1_) and 1.155 Mg m^-3^ (orthorhombic, P2_1_2_1_2_1_).

### Experimental

We have obtained the orthorhombic modification of (I) by recrystallization from chloroform-acetonitrile (1:1) at room temperature. The spectroscopic data are identical to those of monoclinic phase.

### Refinement

In the absence of anomalous scatterers, 3871 Friedel pairs were merged. *PLATON* (Spek, 2009) reports a solvent accessible voids of total area 289.5 Å^3^ in the structure. However, the low residual electron density does not suggest additional solvent in the structure. This was confirmed using the SQUEEZE procedure (Spek, 2009). All H atoms were refined as riding on their parent atoms, with distances of 0.82 (OH), 0.98 (CH), 0.97 (CH_2_) and 0.96 (CH_3_) Å from the parent C and O atoms, with *U*_iso_(H) = 1.2*U*_eq_(C, O) or 1.5*U*_eq_(C).

### Crystal data

**Table:**

C_20_H_30_O_4_	*F*(000) = 1456
*M**_r_* = 334.44	*D*_x_ = 1.155 Mg m^−^^3^
Orthorhombic, *P*2_1_2_1_2_1_	Mo *K*α radiation, λ = 0.71073 Å
Hall symbol: P 2ac 2ab	Cell parameters from 5298 reflections
*a* = 7.4160 (15) Å	θ = 1.3–28.6°
*b* = 19.374 (4) Å	µ = 0.08 mm^−^^1^
*c* = 26.767 (5) Å	*T* = 293 K
*V* = 3845.8 (13) Å^3^	Prism, colourless
*Z* = 8	0.18 × 0.15 × 0.08 mm

### Data collection

**Table:**

Nonius KappaCCD area-detector diffractometer	4452 reflections with *I* > 2σ(*I*)
Radiation source: fine-focus sealed tube	*R*_int_ = 0.08
graphite	θ_max_ = 28.6°, θ_min_ = 1.3°
φ scans, and ω scans with κ offsets	*h* = −9→9
43215 measured reflections	*k* = −26→26
5387 independent reflections	*l* = −35→35

### Refinement

**Table:**

Refinement on *F*^2^	Secondary atom site location: difference Fourier map
Least-squares matrix: full	Hydrogen site location: inferred from neighbouring sites
*R*[*F*^2^ > 2σ(*F*^2^)] = 0.091	H-atom parameters constrained
*wR*(*F*^2^) = 0.224	*w* = 1/[σ^2^(*F*_o_^2^) + (0.1087*P*)^2^ + 1.0561*P*] where *P* = (*F*_o_^2^ + 2*F*_c_^2^)/3
*S* = 1.25	(Δ/σ)_max_ < 0.001
5387 reflections	Δρ_max_ = 0.45 e Å^−^^3^
444 parameters	Δρ_min_ = −0.48 e Å^−^^3^
0 restraints	Extinction correction: *SHELXL97* (Sheldrick, 2008)
Primary atom site location: structure-invariant direct methods	Extinction coefficient: 0.038 (4)

### Special details

**Table:**

Geometry. All s.u.'s (except the s.u. in the dihedral angle between two l.s. planes) are estimated using the full covariance matrix. The cell s.u.'s are taken into account individually in the estimation of s.u.'s in distances, angles and torsion angles; correlations between s.u.'s in cell parameters are only used when they are defined by crystal symmetry. An approximate (isotropic) treatment of cell s.u.'s is used for estimating s.u.'s involving l.s. planes.
Refinement. Refinement of *F*^2^ against ALL reflections. The weighted *R*-factor *wR* and goodness of fit *S* are based on *F*^2^, conventional *R*-factors *R* are based on *F*, with *F* set to zero for negative *F*^2^. The threshold expression of *F*^2^ > 2σ(*F*^2^) is used only for calculating *R*-factors(gt) *etc*. and is not relevant to the choice of reflections for refinement. *R*-factors based on *F*^2^ are statistically about twice as large as those based on *F*, and *R*- factors based on ALL data will be even larger.

### Fractional atomic coordinates and isotropic or equivalent isotropic displacement parameters (Å^2^)

**Table:**

	*x*	*y*	*z*	*U*_iso_*/*U*_eq_	
O1	1.0240 (4)	0.5230 (2)	0.81073 (10)	0.0568 (9)	
O2	0.7577 (4)	0.5035 (2)	0.84418 (9)	0.0526 (8)	
H2	0.8148	0.5086	0.8701	0.079*	
O3	1.0415 (5)	0.47601 (16)	0.62047 (10)	0.0487 (7)	
O4	0.9156 (4)	0.42951 (17)	0.59483 (11)	0.0548 (8)	
C1	0.9999 (6)	0.5802 (2)	0.70754 (15)	0.0400 (9)	
H1A	1.1148	0.5759	0.7246	0.048*	
H1B	1.0218	0.5925	0.6729	0.048*	
C2	0.8811 (6)	0.6343 (2)	0.73306 (16)	0.0443 (10)	
H2A	0.8567	0.6722	0.7104	0.053*	
H2B	0.9411	0.6525	0.7624	0.053*	
C3	0.7038 (5)	0.59812 (19)	0.74797 (14)	0.0353 (8)	
H3	0.6258	0.5986	0.7184	0.042*	
C4	0.6015 (6)	0.6359 (2)	0.78963 (15)	0.0432 (9)	
H4	0.6791	0.6373	0.8192	0.052*	
C5	0.7641 (5)	0.52173 (18)	0.75618 (12)	0.0302 (7)	
C6	0.6212 (5)	0.4648 (2)	0.75110 (14)	0.0384 (9)	
H6A	0.5356	0.4679	0.7784	0.046*	
H6B	0.5558	0.4705	0.72	0.046*	
C7	0.7130 (7)	0.3948 (2)	0.75195 (16)	0.0447 (10)	
H7A	0.621	0.36	0.7467	0.054*	
H7B	0.7608	0.3878	0.7853	0.054*	
C8	0.8680 (6)	0.38068 (19)	0.71418 (15)	0.0400 (9)	
C9	0.9969 (5)	0.44437 (19)	0.70991 (13)	0.0347 (8)	
H9	1.0742	0.4438	0.7396	0.042*	
C10	0.8922 (5)	0.51262 (18)	0.71109 (12)	0.0307 (7)	
H10	0.8142	0.5119	0.6815	0.037*	
C11	1.1245 (5)	0.4450 (2)	0.66369 (14)	0.0409 (9)	
H11	1.2274	0.4742	0.6726	0.049*	
C12	1.2001 (7)	0.3765 (3)	0.64918 (17)	0.0552 (12)	
H12	1.3186	0.3646	0.6565	0.066*	
C13	1.0920 (8)	0.3340 (2)	0.62570 (16)	0.0522 (12)	
C14	0.9036 (7)	0.3622 (2)	0.61696 (16)	0.0494 (11)	
H14	0.8456	0.332	0.5923	0.059*	
C15	0.7823 (6)	0.3629 (2)	0.66327 (16)	0.0469 (10)	
H15A	0.7272	0.3177	0.6662	0.056*	
H15B	0.6859	0.3957	0.6573	0.056*	
C16	1.1342 (10)	0.2621 (3)	0.6100 (2)	0.0788 (18)	
H16A	1.2567	0.2514	0.6187	0.118*	
H16B	1.1189	0.2579	0.5745	0.118*	
H16C	1.0544	0.2307	0.6267	0.118*	
C17	0.9659 (9)	0.3176 (2)	0.7367 (2)	0.0638 (14)	
H17A	1.0523	0.3003	0.7132	0.096*	
H17B	0.8795	0.2822	0.7442	0.096*	
H17C	1.0267	0.3311	0.7668	0.096*	
C18	0.5621 (9)	0.7103 (3)	0.7738 (2)	0.0706 (15)	
H18A	0.4991	0.7337	0.8001	0.106*	
H18B	0.489	0.7102	0.7442	0.106*	
H18C	0.6735	0.7338	0.7671	0.106*	
C19	0.4256 (6)	0.6015 (3)	0.80408 (19)	0.0585 (12)	
H19A	0.4502	0.5573	0.8188	0.088*	
H19B	0.3522	0.5955	0.7749	0.088*	
H19C	0.3629	0.6299	0.8278	0.088*	
C20	0.8616 (5)	0.51600 (19)	0.80626 (13)	0.0331 (8)	
O1A	0.5588 (4)	0.48787 (19)	0.43116 (10)	0.0542 (8)	
O2A	0.2933 (4)	0.50197 (18)	0.39526 (9)	0.0485 (8)	
H2AA	0.3511	0.4933	0.3699	0.073*	
O3A	0.5334 (5)	0.54458 (17)	0.62191 (10)	0.0529 (8)	
O4A	0.3982 (5)	0.59001 (18)	0.64451 (12)	0.0583 (9)	
C1A	0.5303 (6)	0.4365 (2)	0.53523 (15)	0.0413 (9)	
H1A1	0.646	0.4431	0.519	0.05*	
H1A2	0.5503	0.4257	0.5702	0.05*	
C2A	0.4232 (6)	0.3791 (2)	0.50961 (17)	0.0483 (10)	
H2A1	0.4017	0.3415	0.5328	0.058*	
H2A2	0.4899	0.3613	0.4812	0.058*	
C3A	0.2422 (5)	0.41043 (19)	0.49212 (13)	0.0348 (8)	
H3A	0.1601	0.409	0.5208	0.042*	
C4A	0.1512 (6)	0.3704 (2)	0.44994 (15)	0.0428 (9)	
H4A	0.2264	0.3751	0.42	0.051*	
C5A	0.2929 (5)	0.48839 (19)	0.48342 (12)	0.0293 (7)	
C6A	0.1421 (5)	0.5416 (2)	0.48575 (14)	0.0375 (8)	
H6A1	0.0605	0.5352	0.4578	0.045*	
H6A2	0.0742	0.536	0.5165	0.045*	
C7A	0.2246 (6)	0.6134 (2)	0.48375 (15)	0.0441 (10)	
H7A1	0.1275	0.6467	0.4868	0.053*	
H7A2	0.2773	0.6196	0.4509	0.053*	
C8A	0.3718 (6)	0.6322 (2)	0.52339 (15)	0.0414 (9)	
C9A	0.5069 (5)	0.57135 (19)	0.53126 (13)	0.0341 (8)	
H9A	0.5908	0.5723	0.503	0.041*	
C10A	0.4126 (5)	0.50107 (18)	0.52985 (11)	0.0305 (7)	
H10A	0.3306	0.5006	0.5586	0.037*	
C11A	0.6238 (6)	0.5749 (2)	0.57973 (15)	0.0461 (10)	
H11A	0.7315	0.5468	0.5735	0.055*	
C12A	0.6884 (7)	0.6452 (3)	0.59389 (17)	0.0612 (14)	
H12A	0.8067	0.6589	0.588	0.073*	
C13A	0.5714 (9)	0.6868 (3)	0.61497 (18)	0.0599 (14)	
C14A	0.3857 (7)	0.6562 (2)	0.62047 (17)	0.0519 (11)	
H14A	0.3192	0.6864	0.6433	0.062*	
C15A	0.2736 (7)	0.6515 (2)	0.57253 (17)	0.0487 (10)	
H15C	0.2152	0.6957	0.5675	0.058*	
H15D	0.1791	0.6177	0.578	0.058*	
C16A	0.6059 (11)	0.7598 (3)	0.6313 (2)	0.089 (2)	
H16D	0.7287	0.7719	0.6241	0.133*	
H16E	0.5846	0.7637	0.6666	0.133*	
H16F	0.5263	0.7904	0.6137	0.133*	
C17A	0.4677 (9)	0.6961 (2)	0.5010 (2)	0.0645 (14)	
H17D	0.3793	0.7299	0.4916	0.097*	
H17E	0.5354	0.6825	0.4721	0.097*	
H17F	0.5477	0.7155	0.5254	0.097*	
C18A	0.1365 (10)	0.2934 (3)	0.4622 (3)	0.0786 (18)	
H18D	0.0555	0.2872	0.4898	0.118*	
H18E	0.2533	0.2758	0.4709	0.118*	
H18F	0.0913	0.2691	0.4336	0.118*	
C19A	−0.0363 (7)	0.3971 (3)	0.4373 (2)	0.0663 (14)	
H19D	−0.028	0.4439	0.4259	0.099*	
H19E	−0.111	0.3951	0.4665	0.099*	
H19F	−0.0881	0.3689	0.4115	0.099*	
C20A	0.3954 (5)	0.49324 (19)	0.43429 (12)	0.0337 (8)	

### Atomic displacement parameters (Å^2^)

**Table:**

	*U*^11^	*U*^22^	*U*^33^	*U*^12^	*U*^13^	*U*^23^
O1	0.0367 (16)	0.107 (3)	0.0270 (13)	−0.0195 (17)	−0.0079 (12)	0.0018 (15)
O2	0.0400 (14)	0.095 (2)	0.0229 (12)	−0.0030 (16)	0.0021 (11)	0.0077 (15)
O3	0.0585 (18)	0.0556 (17)	0.0320 (14)	−0.0015 (15)	0.0073 (14)	0.0055 (12)
O4	0.0499 (18)	0.070 (2)	0.0448 (17)	0.0049 (16)	−0.0087 (15)	0.0053 (15)
C1	0.040 (2)	0.048 (2)	0.0325 (19)	−0.0154 (18)	0.0021 (17)	0.0026 (16)
C2	0.052 (2)	0.042 (2)	0.039 (2)	−0.010 (2)	0.0065 (19)	−0.0013 (17)
C3	0.037 (2)	0.0415 (19)	0.0277 (17)	−0.0020 (16)	−0.0024 (16)	−0.0018 (15)
C4	0.042 (2)	0.052 (2)	0.0352 (19)	0.005 (2)	−0.0042 (18)	−0.0091 (17)
C5	0.0302 (17)	0.0405 (18)	0.0200 (14)	−0.0088 (15)	−0.0019 (13)	0.0006 (13)
C6	0.0318 (19)	0.054 (2)	0.0297 (17)	−0.0150 (17)	0.0011 (15)	−0.0033 (16)
C7	0.056 (3)	0.039 (2)	0.038 (2)	−0.0126 (19)	0.008 (2)	0.0039 (16)
C8	0.048 (2)	0.0360 (18)	0.036 (2)	−0.0017 (18)	0.0025 (18)	0.0026 (15)
C9	0.0317 (18)	0.047 (2)	0.0253 (16)	0.0014 (16)	−0.0027 (15)	0.0022 (15)
C10	0.0298 (16)	0.0423 (18)	0.0200 (14)	−0.0042 (15)	0.0000 (13)	0.0012 (13)
C11	0.0316 (19)	0.059 (2)	0.0326 (19)	−0.0009 (18)	0.0009 (16)	−0.0007 (17)
C12	0.046 (2)	0.077 (3)	0.043 (2)	0.020 (3)	0.003 (2)	−0.004 (2)
C13	0.066 (3)	0.052 (2)	0.038 (2)	0.015 (2)	0.007 (2)	−0.0054 (19)
C14	0.055 (3)	0.055 (2)	0.038 (2)	0.003 (2)	−0.003 (2)	−0.0140 (19)
C15	0.050 (2)	0.048 (2)	0.043 (2)	−0.007 (2)	0.0003 (19)	−0.0107 (18)
C16	0.109 (5)	0.064 (3)	0.064 (3)	0.032 (3)	0.005 (4)	−0.012 (3)
C17	0.085 (4)	0.054 (3)	0.053 (3)	0.012 (3)	0.007 (3)	0.014 (2)
C18	0.077 (4)	0.056 (3)	0.079 (4)	0.015 (3)	0.012 (3)	−0.009 (3)
C19	0.039 (2)	0.087 (3)	0.049 (3)	0.000 (2)	0.009 (2)	−0.009 (2)
C20	0.0361 (19)	0.0412 (19)	0.0221 (15)	−0.0036 (16)	−0.0036 (14)	−0.0019 (14)
O1A	0.0362 (15)	0.101 (2)	0.0252 (12)	0.0121 (17)	0.0038 (11)	−0.0004 (15)
O2A	0.0437 (15)	0.083 (2)	0.0188 (11)	−0.0009 (17)	−0.0032 (11)	0.0062 (14)
O3A	0.066 (2)	0.0612 (18)	0.0314 (14)	−0.0056 (17)	−0.0109 (15)	0.0053 (13)
O4A	0.062 (2)	0.069 (2)	0.0445 (17)	−0.0099 (18)	0.0064 (16)	0.0081 (15)
C1A	0.039 (2)	0.052 (2)	0.0333 (19)	0.0154 (18)	−0.0056 (17)	0.0048 (16)
C2A	0.055 (3)	0.045 (2)	0.044 (2)	0.014 (2)	−0.010 (2)	0.0045 (18)
C3A	0.040 (2)	0.0404 (18)	0.0242 (16)	0.0053 (17)	0.0016 (15)	0.0046 (14)
C4A	0.050 (2)	0.044 (2)	0.034 (2)	−0.0052 (19)	0.0020 (18)	−0.0026 (17)
C5A	0.0272 (16)	0.0405 (17)	0.0200 (14)	0.0035 (15)	−0.0025 (13)	0.0029 (13)
C6A	0.0330 (19)	0.046 (2)	0.0333 (19)	0.0085 (17)	−0.0058 (16)	−0.0012 (16)
C7A	0.056 (2)	0.041 (2)	0.035 (2)	0.017 (2)	−0.008 (2)	0.0055 (16)
C8A	0.051 (2)	0.0385 (19)	0.0351 (19)	−0.0003 (19)	−0.0012 (18)	0.0026 (16)
C9A	0.0313 (18)	0.047 (2)	0.0237 (16)	−0.0014 (16)	0.0008 (14)	0.0013 (14)
C10A	0.0313 (17)	0.0421 (18)	0.0180 (14)	0.0030 (15)	−0.0028 (13)	0.0004 (13)
C11A	0.038 (2)	0.067 (3)	0.033 (2)	−0.003 (2)	−0.0047 (18)	−0.0005 (18)
C12A	0.055 (3)	0.091 (4)	0.038 (2)	−0.031 (3)	−0.005 (2)	0.002 (2)
C13A	0.081 (4)	0.065 (3)	0.034 (2)	−0.027 (3)	−0.005 (2)	−0.003 (2)
C14A	0.061 (3)	0.056 (2)	0.039 (2)	−0.003 (2)	0.005 (2)	−0.0095 (19)
C15A	0.052 (2)	0.045 (2)	0.049 (2)	0.008 (2)	0.003 (2)	−0.0054 (19)
C16A	0.134 (6)	0.070 (4)	0.061 (3)	−0.041 (4)	−0.006 (4)	−0.007 (3)
C17A	0.087 (4)	0.050 (3)	0.056 (3)	−0.013 (3)	−0.007 (3)	0.016 (2)
C18A	0.098 (4)	0.050 (3)	0.088 (4)	−0.019 (3)	−0.019 (4)	0.000 (3)
C19A	0.054 (3)	0.076 (3)	0.068 (3)	−0.008 (3)	−0.020 (3)	−0.010 (3)
C20A	0.0375 (19)	0.0413 (18)	0.0223 (15)	0.0019 (16)	0.0004 (14)	0.0029 (14)

### Geometric parameters (Å, °)

**Table:**

O1—C20	1.218 (5)	O1A—C20A	1.220 (5)
O2—C20	1.298 (5)	O2A—C20A	1.301 (4)
O2—H2	0.82	O2A—H2AA	0.82
O3—C11	1.442 (5)	O3A—C11A	1.438 (5)
O3—O4	1.468 (4)	O3A—O4A	1.465 (5)
O4—C14	1.435 (6)	O4A—C14A	1.437 (6)
C1—C2	1.530 (6)	C1A—C2A	1.529 (6)
C1—C10	1.537 (5)	C1A—C10A	1.532 (5)
C1—H1A	0.97	C1A—H1A1	0.97
C1—H1B	0.97	C1A—H1A2	0.97
C2—C3	1.542 (6)	C2A—C3A	1.546 (6)
C2—H2A	0.97	C2A—H2A1	0.97
C2—H2B	0.97	C2A—H2A2	0.97
C3—C4	1.535 (5)	C3A—C4A	1.527 (5)
C3—C5	1.562 (5)	C3A—C5A	1.574 (5)
C3—H3	0.98	C3A—H3A	0.98
C4—C19	1.515 (6)	C4A—C19A	1.522 (7)
C4—C18	1.531 (7)	C4A—C18A	1.530 (6)
C4—H4	0.98	C4A—H4A	0.98
C5—C20	1.527 (5)	C5A—C20A	1.522 (5)
C5—C6	1.536 (5)	C5A—C6A	1.522 (5)
C5—C10	1.546 (5)	C5A—C10A	1.547 (4)
C6—C7	1.517 (6)	C6A—C7A	1.521 (6)
C6—H6A	0.97	C6A—H6A1	0.97
C6—H6B	0.97	C6A—H6A2	0.97
C7—C8	1.555 (6)	C7A—C8A	1.565 (6)
C7—H7A	0.97	C7A—H7A1	0.97
C7—H7B	0.97	C7A—H7A2	0.97
C8—C15	1.543 (6)	C8A—C17A	1.548 (6)
C8—C17	1.545 (6)	C8A—C15A	1.549 (6)
C8—C9	1.565 (5)	C8A—C9A	1.561 (5)
C9—C10	1.534 (5)	C9A—C10A	1.531 (5)
C9—C11	1.558 (5)	C9A—C11A	1.562 (5)
C9—H9	0.98	C9A—H9A	0.98
C10—H10	0.98	C10A—H10A	0.98
C11—C12	1.491 (6)	C11A—C12A	1.493 (7)
C11—H11	0.98	C11A—H11A	0.98
C12—C13	1.310 (7)	C12A—C13A	1.312 (8)
C12—H12	0.93	C12A—H12A	0.93
C13—C16	1.488 (6)	C13A—C16A	1.502 (7)
C13—C14	1.519 (7)	C13A—C14A	1.507 (8)
C14—C15	1.531 (6)	C14A—C15A	1.532 (7)
C14—H14	0.98	C14A—H14A	0.98
C15—H15A	0.97	C15A—H15C	0.97
C15—H15B	0.97	C15A—H15D	0.97
C16—H16A	0.96	C16A—H16D	0.96
C16—H16B	0.96	C16A—H16E	0.96
C16—H16C	0.96	C16A—H16F	0.96
C17—H17A	0.96	C17A—H17D	0.96
C17—H17B	0.96	C17A—H17E	0.96
C17—H17C	0.96	C17A—H17F	0.96
C18—H18A	0.96	C18A—H18D	0.96
C18—H18B	0.96	C18A—H18E	0.96
C18—H18C	0.96	C18A—H18F	0.96
C19—H19A	0.96	C19A—H19D	0.96
C19—H19B	0.96	C19A—H19E	0.96
C19—H19C	0.96	C19A—H19F	0.96
			
C20—O2—H2	109.5	C20A—O2A—H2AA	109.5
C11—O3—O4	113.0 (3)	C11A—O3A—O4A	113.5 (3)
C14—O4—O3	113.8 (3)	C14A—O4A—O3A	113.3 (3)
C2—C1—C10	104.8 (3)	C2A—C1A—C10A	104.8 (3)
C2—C1—H1A	110.8	C2A—C1A—H1A1	110.8
C10—C1—H1A	110.8	C10A—C1A—H1A1	110.8
C2—C1—H1B	110.8	C2A—C1A—H1A2	110.8
C10—C1—H1B	110.8	C10A—C1A—H1A2	110.8
H1A—C1—H1B	108.9	H1A1—C1A—H1A2	108.9
C1—C2—C3	107.2 (3)	C1A—C2A—C3A	107.5 (3)
C1—C2—H2A	110.3	C1A—C2A—H2A1	110.2
C3—C2—H2A	110.3	C3A—C2A—H2A1	110.2
C1—C2—H2B	110.3	C1A—C2A—H2A2	110.2
C3—C2—H2B	110.3	C3A—C2A—H2A2	110.2
H2A—C2—H2B	108.5	H2A1—C2A—H2A2	108.5
C4—C3—C2	113.1 (3)	C4A—C3A—C2A	114.1 (3)
C4—C3—C5	119.4 (3)	C4A—C3A—C5A	118.9 (3)
C2—C3—C5	102.8 (3)	C2A—C3A—C5A	102.4 (3)
C4—C3—H3	106.9	C4A—C3A—H3A	106.9
C2—C3—H3	106.9	C2A—C3A—H3A	106.9
C5—C3—H3	106.9	C5A—C3A—H3A	106.9
C19—C4—C18	108.7 (4)	C19A—C4A—C3A	113.3 (4)
C19—C4—C3	113.7 (4)	C19A—C4A—C18A	108.2 (4)
C18—C4—C3	110.0 (4)	C3A—C4A—C18A	111.6 (4)
C19—C4—H4	108.1	C19A—C4A—H4A	107.8
C18—C4—H4	108.1	C3A—C4A—H4A	107.8
C3—C4—H4	108.1	C18A—C4A—H4A	107.8
C20—C5—C6	110.6 (3)	C20A—C5A—C6A	111.1 (3)
C20—C5—C10	112.7 (3)	C20A—C5A—C10A	113.4 (3)
C6—C5—C10	105.8 (3)	C6A—C5A—C10A	106.3 (3)
C20—C5—C3	109.2 (3)	C20A—C5A—C3A	107.8 (3)
C6—C5—C3	118.1 (3)	C6A—C5A—C3A	117.9 (3)
C10—C5—C3	100.0 (3)	C10A—C5A—C3A	99.8 (3)
C7—C6—C5	109.3 (3)	C7A—C6A—C5A	108.8 (3)
C7—C6—H6A	109.8	C7A—C6A—H6A1	109.9
C5—C6—H6A	109.8	C5A—C6A—H6A1	109.9
C7—C6—H6B	109.8	C7A—C6A—H6A2	109.9
C5—C6—H6B	109.8	C5A—C6A—H6A2	109.9
H6A—C6—H6B	108.3	H6A1—C6A—H6A2	108.3
C6—C7—C8	118.6 (3)	C6A—C7A—C8A	118.0 (3)
C6—C7—H7A	107.7	C6A—C7A—H7A1	107.8
C8—C7—H7A	107.7	C8A—C7A—H7A1	107.8
C6—C7—H7B	107.7	C6A—C7A—H7A2	107.8
C8—C7—H7B	107.7	C8A—C7A—H7A2	107.8
H7A—C7—H7B	107.1	H7A1—C7A—H7A2	107.1
C15—C8—C17	111.2 (4)	C17A—C8A—C15A	110.6 (4)
C15—C8—C7	108.0 (4)	C17A—C8A—C9A	111.2 (4)
C17—C8—C7	103.4 (4)	C15A—C8A—C9A	111.7 (3)
C15—C8—C9	111.3 (3)	C17A—C8A—C7A	104.1 (3)
C17—C8—C9	111.4 (4)	C15A—C8A—C7A	107.7 (4)
C7—C8—C9	111.1 (3)	C9A—C8A—C7A	111.3 (3)
C10—C9—C11	108.5 (3)	C10A—C9A—C8A	112.1 (3)
C10—C9—C8	111.6 (3)	C10A—C9A—C11A	108.2 (3)
C11—C9—C8	115.8 (3)	C8A—C9A—C11A	115.8 (3)
C10—C9—H9	106.8	C10A—C9A—H9A	106.8
C11—C9—H9	106.8	C8A—C9A—H9A	106.8
C8—C9—H9	106.8	C11A—C9A—H9A	106.8
C9—C10—C1	118.0 (3)	C9A—C10A—C1A	117.6 (3)
C9—C10—C5	115.2 (3)	C9A—C10A—C5A	115.0 (3)
C1—C10—C5	105.7 (3)	C1A—C10A—C5A	105.8 (3)
C9—C10—H10	105.6	C9A—C10A—H10A	105.8
C1—C10—H10	105.6	C1A—C10A—H10A	105.8
C5—C10—H10	105.6	C5A—C10A—H10A	105.8
O3—C11—C12	108.8 (3)	O3A—C11A—C12A	108.8 (4)
O3—C11—C9	112.4 (3)	O3A—C11A—C9A	112.1 (3)
C12—C11—C9	115.4 (4)	C12A—C11A—C9A	115.4 (4)
O3—C11—H11	106.6	O3A—C11A—H11A	106.7
C12—C11—H11	106.6	C12A—C11A—H11A	106.7
C9—C11—H11	106.6	C9A—C11A—H11A	106.7
C13—C12—C11	117.0 (4)	C13A—C12A—C11A	117.2 (5)
C13—C12—H12	121.5	C13A—C12A—H12A	121.4
C11—C12—H12	121.5	C11A—C12A—H12A	121.4
C12—C13—C16	126.5 (5)	C12A—C13A—C16A	126.2 (6)
C12—C13—C14	114.2 (4)	C12A—C13A—C14A	113.9 (4)
C16—C13—C14	119.2 (5)	C16A—C13A—C14A	119.8 (6)
O4—C14—C13	109.5 (4)	O4A—C14A—C13A	109.6 (4)
O4—C14—C15	111.3 (4)	O4A—C14A—C15A	110.9 (4)
C13—C14—C15	114.7 (4)	C13A—C14A—C15A	116.0 (4)
O4—C14—H14	107	O4A—C14A—H14A	106.6
C13—C14—H14	107	C13A—C14A—H14A	106.6
C15—C14—H14	107	C15A—C14A—H14A	106.6
C14—C15—C8	118.4 (4)	C14A—C15A—C8A	118.1 (4)
C14—C15—H15A	107.7	C14A—C15A—H15C	107.8
C8—C15—H15A	107.7	C8A—C15A—H15C	107.8
C14—C15—H15B	107.7	C14A—C15A—H15D	107.8
C8—C15—H15B	107.7	C8A—C15A—H15D	107.8
H15A—C15—H15B	107.1	H15C—C15A—H15D	107.1
C13—C16—H16A	109.5	C13A—C16A—H16D	109.5
C13—C16—H16B	109.5	C13A—C16A—H16E	109.5
H16A—C16—H16B	109.5	H16D—C16A—H16E	109.5
C13—C16—H16C	109.5	C13A—C16A—H16F	109.5
H16A—C16—H16C	109.5	H16D—C16A—H16F	109.5
H16B—C16—H16C	109.5	H16E—C16A—H16F	109.5
C8—C17—H17A	109.5	C8A—C17A—H17D	109.5
C8—C17—H17B	109.5	C8A—C17A—H17E	109.5
H17A—C17—H17B	109.5	H17D—C17A—H17E	109.5
C8—C17—H17C	109.5	C8A—C17A—H17F	109.5
H17A—C17—H17C	109.5	H17D—C17A—H17F	109.5
H17B—C17—H17C	109.5	H17E—C17A—H17F	109.5
C4—C18—H18A	109.5	C4A—C18A—H18D	109.5
C4—C18—H18B	109.5	C4A—C18A—H18E	109.5
H18A—C18—H18B	109.5	H18D—C18A—H18E	109.5
C4—C18—H18C	109.5	C4A—C18A—H18F	109.5
H18A—C18—H18C	109.5	H18D—C18A—H18F	109.5
H18B—C18—H18C	109.5	H18E—C18A—H18F	109.5
C4—C19—H19A	109.5	C4A—C19A—H19D	109.5
C4—C19—H19B	109.5	C4A—C19A—H19E	109.5
H19A—C19—H19B	109.5	H19D—C19A—H19E	109.5
C4—C19—H19C	109.5	C4A—C19A—H19F	109.5
H19A—C19—H19C	109.5	H19D—C19A—H19F	109.5
H19B—C19—H19C	109.5	H19E—C19A—H19F	109.5
O1—C20—O2	122.1 (3)	O1A—C20A—O2A	122.3 (3)
O1—C20—C5	123.1 (3)	O1A—C20A—C5A	123.4 (3)
O2—C20—C5	114.8 (3)	O2A—C20A—C5A	114.3 (3)
			
C11—O3—O4—C14	−1.5 (4)	C11A—O3A—O4A—C14A	−1.1 (5)
C10—C1—C2—C3	−3.2 (4)	C10A—C1A—C2A—C3A	−2.6 (4)
C1—C2—C3—C4	158.9 (3)	C1A—C2A—C3A—C4A	158.1 (3)
C1—C2—C3—C5	28.7 (4)	C1A—C2A—C3A—C5A	28.3 (4)
C2—C3—C4—C19	179.1 (4)	C2A—C3A—C4A—C19A	173.4 (4)
C5—C3—C4—C19	−59.6 (5)	C5A—C3A—C4A—C19A	−65.6 (5)
C2—C3—C4—C18	57.0 (5)	C2A—C3A—C4A—C18A	50.9 (5)
C5—C3—C4—C18	178.2 (4)	C5A—C3A—C4A—C18A	171.9 (4)
C4—C3—C5—C20	−49.9 (4)	C4A—C3A—C5A—C20A	−50.0 (4)
C2—C3—C5—C20	76.4 (3)	C2A—C3A—C5A—C20A	76.7 (3)
C4—C3—C5—C6	77.6 (4)	C4A—C3A—C5A—C6A	76.8 (4)
C2—C3—C5—C6	−156.2 (3)	C2A—C3A—C5A—C6A	−156.5 (3)
C4—C3—C5—C10	−168.3 (3)	C4A—C3A—C5A—C10A	−168.7 (3)
C2—C3—C5—C10	−42.1 (3)	C2A—C3A—C5A—C10A	−42.0 (3)
C20—C5—C6—C7	−62.5 (4)	C20A—C5A—C6A—C7A	−62.8 (4)
C10—C5—C6—C7	59.8 (4)	C10A—C5A—C6A—C7A	61.1 (4)
C3—C5—C6—C7	170.7 (3)	C3A—C5A—C6A—C7A	172.0 (3)
C5—C6—C7—C8	−54.2 (5)	C5A—C6A—C7A—C8A	−55.1 (5)
C6—C7—C8—C15	−79.9 (4)	C6A—C7A—C8A—C17A	162.2 (4)
C6—C7—C8—C17	162.2 (4)	C6A—C7A—C8A—C15A	−80.3 (4)
C6—C7—C8—C9	42.5 (5)	C6A—C7A—C8A—C9A	42.3 (5)
C15—C8—C9—C10	81.2 (4)	C17A—C8A—C9A—C10A	−153.8 (3)
C17—C8—C9—C10	−154.0 (3)	C15A—C8A—C9A—C10A	82.1 (4)
C7—C8—C9—C10	−39.2 (4)	C7A—C8A—C9A—C10A	−38.2 (4)
C15—C8—C9—C11	−43.6 (5)	C17A—C8A—C9A—C11A	81.4 (4)
C17—C8—C9—C11	81.2 (4)	C15A—C8A—C9A—C11A	−42.7 (5)
C7—C8—C9—C11	−164.0 (3)	C7A—C8A—C9A—C11A	−163.0 (3)
C11—C9—C10—C1	−51.6 (4)	C8A—C9A—C10A—C1A	177.9 (3)
C8—C9—C10—C1	179.7 (3)	C11A—C9A—C10A—C1A	−53.2 (4)
C11—C9—C10—C5	−177.6 (3)	C8A—C9A—C10A—C5A	52.1 (4)
C8—C9—C10—C5	53.6 (4)	C11A—C9A—C10A—C5A	−179.0 (3)
C2—C1—C10—C9	−154.6 (3)	C2A—C1A—C10A—C9A	−155.0 (3)
C2—C1—C10—C5	−24.0 (4)	C2A—C1A—C10A—C5A	−24.9 (4)
C20—C5—C10—C9	57.5 (4)	C20A—C5A—C10A—C9A	58.9 (4)
C6—C5—C10—C9	−63.6 (4)	C6A—C5A—C10A—C9A	−63.6 (4)
C3—C5—C10—C9	173.3 (3)	C3A—C5A—C10A—C9A	173.3 (3)
C20—C5—C10—C1	−74.7 (4)	C20A—C5A—C10A—C1A	−72.8 (4)
C6—C5—C10—C1	164.3 (3)	C6A—C5A—C10A—C1A	164.8 (3)
C3—C5—C10—C1	41.1 (3)	C3A—C5A—C10A—C1A	41.7 (3)
O4—O3—C11—C12	50.5 (4)	O4A—O3A—C11A—C12A	49.7 (4)
O4—O3—C11—C9	−78.6 (4)	O4A—O3A—C11A—C9A	−79.1 (4)
C10—C9—C11—O3	−39.1 (4)	C10A—C9A—C11A—O3A	−40.1 (4)
C8—C9—C11—O3	87.3 (4)	C8A—C9A—C11A—O3A	86.7 (4)
C10—C9—C11—C12	−164.7 (3)	C10A—C9A—C11A—C12A	−165.4 (4)
C8—C9—C11—C12	−38.2 (5)	C8A—C9A—C11A—C12A	−38.6 (5)
O3—C11—C12—C13	−51.0 (5)	O3A—C11A—C12A—C13A	−49.8 (6)
C9—C11—C12—C13	76.3 (5)	C9A—C11A—C12A—C13A	77.1 (5)
C11—C12—C13—C16	−176.6 (5)	C11A—C12A—C13A—C16A	−178.9 (5)
C11—C12—C13—C14	0.1 (6)	C11A—C12A—C13A—C14A	−1.2 (6)
O3—O4—C14—C13	−48.5 (4)	O3A—O4A—C14A—C13A	−49.5 (5)
O3—O4—C14—C15	79.4 (4)	O3A—O4A—C14A—C15A	79.8 (5)
C12—C13—C14—O4	50.4 (5)	C12A—C13A—C14A—O4A	51.7 (5)
C16—C13—C14—O4	−132.7 (4)	C16A—C13A—C14A—O4A	−130.5 (5)
C12—C13—C14—C15	−75.6 (5)	C12A—C13A—C14A—C15A	−74.8 (6)
C16—C13—C14—C15	101.3 (5)	C16A—C13A—C14A—C15A	102.9 (5)
O4—C14—C15—C8	−87.5 (5)	O4A—C14A—C15A—C8A	−87.7 (5)
C13—C14—C15—C8	37.5 (6)	C13A—C14A—C15A—C8A	38.2 (6)
C17—C8—C15—C14	−80.3 (5)	C17A—C8A—C15A—C14A	−80.4 (5)
C7—C8—C15—C14	166.9 (4)	C9A—C8A—C15A—C14A	44.0 (5)
C9—C8—C15—C14	44.6 (5)	C7A—C8A—C15A—C14A	166.5 (4)
C6—C5—C20—O1	137.6 (4)	C6A—C5A—C20A—O1A	139.5 (4)
C10—C5—C20—O1	19.4 (5)	C10A—C5A—C20A—O1A	19.8 (6)
C3—C5—C20—O1	−90.8 (5)	C3A—C5A—C20A—O1A	−89.8 (5)
C6—C5—C20—O2	−42.9 (5)	C6A—C5A—C20A—O2A	−42.1 (4)
C10—C5—C20—O2	−161.2 (3)	C10A—C5A—C20A—O2A	−161.9 (3)
C3—C5—C20—O2	88.6 (4)	C3A—C5A—C20A—O2A	88.6 (4)

### Hydrogen-bond geometry (Å, °)

**Table:**

*D*—H···*A*	*D*—H	H···*A*	*D*···*A*	*D*—H···*A*
O2A—H2AA···O1^i^	0.82	1.86	2.681 (4)	177
O2—H2···O1A^ii^	0.82	1.89	2.702 (4)	175

Symmetry codes: (i) −*x*+3/2, −*y*+1, *z*−1/2; (ii) −*x*+3/2, −*y*+1, *z*+1/2.
